# Over-Expression of GUSB Leads to Primary Resistance of Anti-PD1 Therapy in Hepatocellular Carcinoma

**DOI:** 10.3389/fimmu.2022.876048

**Published:** 2022-06-22

**Authors:** Xiangyi Kong, Zhiying Zheng, Guoxin Song, Zihao Zhang, Hanyuan Liu, Junwei Kang, Guoqiang Sun, Guangshun Sun, Tian Huang, Xiao Li, Dawei Rong, Ke Wang, Weiwei Tang, Yongxiang Xia

**Affiliations:** ^1^ Hepatobiliary/Liver Transplantation Center, The First Affiliated Hospital of Nanjing Medical University, Key Laboratory of Living Donor Transplantation, Chinese Academy of Medical Sciences, Nanjing, China; ^2^ Department of Anesthesiology, The First Affiliated Hospital of Nanjing Medical University, Nanjing, China; ^3^ Department of Pathology, The First Affiliated Hospital of Nanjing Medical University, Nanjing, China; ^4^ Department of General Surgery, Nanjing First Hospital, Nanjing Medical University, Nanjing, China

**Keywords:** 289 nanostring panel RNA sequencing, GUSB, PD-1, primary resistance, hepatocellular carcinoma

## Abstract

Immunotherapy treatments, particularly immune checkpoint blockade, can result in benefits in clinical settings. But many pre-clinical and clinical studies have shown that resistance to anti-PD1 therapy frequently occurs, leading to tumor recurrence and treatment failure, including in patients with hepatocellular carcinoma (HCC). In this study, 10 patients with HCC were remedied with anti-PD1, and pre-treatment biopsy samples were sequenced for 289 nanostring panel RNA to compare responsive and non-responsive tumors to identify possible pretreatment biomarkers or targets of anti-PD1 therapeutic responses. Fortunately, the expression of β-Glucuronidase (GUSB) in the non-responding tumors was found to be remarkably higher than that in responding tumors. Results of the cell counting kit 8 (CCK8), 5-ethynyl-2’-deoxyuridine (EdU), transwell, wound healing test, and flow cytometry showed that GUSB facilitated proliferation, invasion, as well as migration of human HCC cells and downregulated PD-L1 expression by promoting miR-513a-5p. Additionally, as a GUSB inhibitor, amoxapine can reduce the progression of human HCC cells, and was an effective treatment for HCC and improved the sensitivity of anti-PD1 therapy. In summary, this study reveals that increased GUSB downregulates PD-L1 expression by promoting miR-513a-5p, leading to primary resistance to anti-PD1 treatment in HCC, and amoxapine enhances the sensitivity of anti-PD1 therapy by inhibiting GUSB, providing a new strategy and method for improving the efficacy of anti-PD1 therapy and bringing new prospects for therapy of HCC.

## Introduction

Hepatocellular carcinoma (HCC) is the most common form of liver cancer and is the third leading cause of cancer-related deaths in the world, but there is a shortage of effective treatment because most patients have reached advanced stages before being diagnosed (1, 2). Therapy with immunotherapy, especially with immune checkpoint blockade therapy, has been shown to bring benefits in clinical settings (3). Anti-PD1 antibodies against the block programmed cell death 1 protein (PD1)/programmed cell death 1 ligand 1 (PD-L1) axis play a major role in inhibiting effector immune cell depletion, leading to impressive advances for treating a variety of advanced malignant neoplasms (3, 4). In spite of the tremendous success, many pre-clinical and clinical studies have shown that anti-PD1 therapy often develops drug resistance, leading to treatment failure and tumor recurrence in patients with HCC (5). Thus, elucidating the underlying anti-PD1 resistance mechanism is vital for improving the prognosis of advanced HCC patients.

Development of PD1/PD-L1 antibody resistance is a complicated, dynamic, and codependent procedure, which is related to many endogenous and exogenous tumor cytokines. According to the time of emergence, resistance is divided into two levels: secondary-level and elementary resistance. Secondary resistance refers to resistance that has effectively controlled tumor development after the initial treatment. Alternatively, initial drug resistance exists before exposure, preventing the drug from blocking tumor progression (6). PD1/PD-L1 resistance is associated with loss of initial and costimulatory signals, the expression level of PD1/PD-L1 on the cell surface, tumor microenvironment, and epigenetic inheritance (7).

In this study, 10 HCC patients received anti-PD1 therapy. To identify potential pretreatment biomarkers or targets of anti-PD1 therapeutic responses, 289 nanostring panels of RNA were sequenced from pretreated biopsy samples from these patients to compare responsive and non-responsive tumors. Fortunately, we found that the expression of β-Glucuronidase (GUSB) in the non-responding tumors was remarkably higher than that in responding tumors. GUSB, as a housekeeping enzyme, is expressed in many tissues and is positively related to lysosomal proteoglycan degradation. By catalyzing the fifth procedure of glycosaminoglycan (GAGs) degradation, it exerts an important role in the degradation of chitosan sulfate and keratin. GUSB is also related to cationic binding and mutual transformation of many metabolites like chlorophyll, gluconate, starch, porphyrin, pentose, and sucrose. Additionally, GUSB exerts an important role in the remodeling of extracellular matrix constitution in physiology and inflammatory situations (8–11). Recently, Bhatt et al. reported that targeted inhibition of GUSB activity enhanced anticancer drug efficacy (12). Sui et al. also summarized the role of GUSB in estrogen reactivation and breast cancer (13). In this research, we discovered that increased GUSB could lead to primary resistance to anti-PD1 therapy by down-regulating PD-L1 expression *via* promoting miR-513a-5p in HCC.

## Materials and Methods

### Nanostring Panel RNA Sequencing

In accordance with the Declaration of Helsinki, we have conducted a notification process on the current research. Ten tumor tissues were collected from the Hepatobiliary/Liver Transplantation Center of the First Affiliated Hospital of Nanjing Medical University for gene expression study before anti-PD1 treatment. Gene expression was studied with the nCounter platform (NanoString Technologies, USA) and transcriptome analysis was performed on the basis of the 289 immune gene panel. Gene expression was tested with the nCounter platform (NanoString Technologies, USA) and transcriptome analysis was performed.

As for the evaluation of tumor necrosis, computed tomography (CT) was used to evaluate the tumor necrosis state before anti-PD1 treatment, and pathological examination was used to evaluate the tumor necrosis state after anti-PD1 treatment, and the calculation method was the product of the maximum long diameter and the maximum vertical diameter.

### Cancer Cell Culture

The Cell Bank of Type Culture Collection (Chinese Academy of Sciences, China) offered mice HCC cells (Hepa1-6) and human HCC cells (Hep-3b and HCC-LM3), cultivated with RPMI 1640 medium (BI, USA) supplemented by 10% fetal bovine serum (FBS) (Gibco, USA) under 37°C in the 5% CO2 chamber. The maintenance was conducted on overall cell lines under the temperature of 37°C within one constant‐temperature incubator with 5% CO2.

### Cell Transfection

We established plasmids of GUSB and lentivirus packaging (Genechem, China) in HCC cells. GUSB was downregulated in human HCC cells (Hep-3b and HCC-LM3) and mouse HCC cells (Hepa1-6) *via* shRNA (Genechem, China). The concentration of Polybrene (Sigma-Aldrich, USA) was increased to 6 µg/ml and a proper quantity of virus was increased to 2 × 10^5^ cells/ml, and fully mixed. After cultivation at 37°C for 4 h, the same quantity of fresh medium was put into the dilute polybrene. sh-GUSB (mice): 5′-GCAGCCCTTCGGGACTTTATT-3′.sh-GUSB(human):5′-ATGTCATTGAAGCTGGAGGGAACTG-3′.

### Cell Proliferation Assay

To detect CCK-8, 10^3^ HCC cells were inoculated into 96-well plates and treated with 10 μl CCK-8 solution (RiboBio, China) at 0, 24, 48, and 72 h, respectively. According to the instructions of the manufacturer (Synergy, USA), cell absorbance was measured at the corresponding time point of 450 nm using a microplate reading element.

With the help of a Cell-Light EdU DNA Cell Proliferation Kit (RiboBio, China), we conducted a 5-ethynyl-2′-deoxyuridine (EdU) assay to evaluate cell proliferation. We put 5 × 10^4^ HCC cells into 24-well plates, then cultured the cells for 24 h, fixed the cell lines with 4% paraformaldehyde after incubation with 50 L for 2 H/L EdU solution. After the agreement with the manufacturer, we put the Apollo dye solution of the cell line and Hearst seal separately. EdU cell lines were collected and calculated under an Olympus FSX100 microscope (Olympus, Japan).

### Transwell Assays

In the upper chamber, Hep-3b and HCC-LM3 cells were seeded with 200 μl serum-free RPMI 1640 medium. The Transwell chamber (Corning, USA) was filled with a matrix mixture (BD Biosciences, USA) for intrusion detection. RPMI 1640 medium and 10% FBS were used as chemokines for HCC cells. After incubation for 24 h, the upper chamber was fixed and stained with crystal violet for 15 min. To visualize the cell process, the cell lines were photographed and counted in three regions.

### Scratch Wound Test

Under the Hep-3b and HCC-LM3 cell confluence, confluence reached about 90% at 48 h post-transfecting process, wounds received the creation using a 200 µl pipette tip, and cells received a rinsing process with a medium for the removal of free-floating cells and debris. The substrate was added and culturing plates were incubated at 37°C. The healed wounds received a survey at different sites. Moreover, by representing scrape lines could be captured.

### Preparation of Antigen-Specific CD8^+^ T Cells

The obtainment of dendritic cells: peripheral blood mononuclear cells (PBMCs) were separated from blood specimens of HCC patients and cultivated in RPMI 1640 with 1% serum for 1 h. IL-4 (50 ng/ml; PeproTech, USA) and granulocyte macrophage colony-stimulating factor (GM-CSF, 100 ng/ml; PeproTech, USA) were put into PMBC and cultivated for 7 days. Next, heat-stimulating human HCC cells (following group: sh-NC, sh-GUSB) were put into and co-cultured for 1 day. Thus, we obtained HCC antigen—loading antigen-presenting cells.

Original CD8^+^ T cells were isolated from PBMCs by magnetic beads using 1 μg/ml CD3 monoclonal antibody (Miltenyi Biotec, Germany), 5 μg/ml CD28 monoclonal antibody, and 20 ng/ml recombinant human interleukin-2 (IL-2; 50 U/ml penicillin purchased from Wobixin Inc., China) was put into the original CD8^+^ T cells. To acquire antigen-responsive CD8^+^ T cells, antigen-loaded HCC antigen-presenting cells were co-cultured with the initial CD8^+^ T cells for 3 days. In the end, antigen-responsive CD8^+^ T cells were co-cultured with HCC cells for 24 h to obtain antigen-specific CD8^+^ T cells.

### Quantitative Reverse Transcription Polymerase Reaction (qRT-PCR)

The total RNA of tissues and cells was separated using TRIzol reagent based on the procedure of the producer (Invitrogen, USA). cDNA was synthesized for PD-L1 and miRNAs using a reversed transcription kit (Takara, Japan) and a RiboBio reversed transcription kit (RiboBio, China). The primers: human PD-L1,5′-TCACTTGGTAATTCTGGGAGC-3′ (forward) and 5′-CTTTGAGTTTGTATCTTGGATGCC-3′ (reverse). GAPDH and U6 were applied to normalized expression levels of PD-L1 and miRNAs before the calculations.

### Western Blotting

Proteins were extracted from the cell using RIPA buffer solution (Sigma-Aldrich, USA), dissolved with SDS-polyacrylamide gel, and transferred to PVDF membranes (Wobixin Inc., China). Main antibodies (Abcam, UK) against PD-L1 were applied. Using peroxidase coupled secondary antibody (CST, Sigma-Aldrich, USA), enhanced chemiluminescence method (ECL; American Hot Fisher).

### Immunohistochemistry

Deparaffinizing and rehydrating processes were conducted for sections with the embedment of paraffin. Approximately 3% hydrogen peroxide was employed to block the peroxidase activity. Under 4°C, sections were incubated throughout the night with primary antibody (CD8, Ki67, PD1, PD-L1, Abcam, UK). Then, the second level of biotinylated antibody was adopted to treat the tissue parts and cultured using the peroxidase complex of streptavidin-horseradish (Santa Cruz Biotechnology Inc., USA).

### Mice Model

The animal management committee of Nanjing Medical University has approved animal experiments, and all experimental steps and animal care follow the institutional ethical guidance for animal-related experimental procedures.

Mice model Part i: The injection of Hepa1-6 cells was made into five-week old male C57BL/6 mice. Carcinoma transplanted tumor model mice fell into four groups, i.e., sh-NC, sh-GUSB, sh-NC+ anti-PD1 (Bio X cell, USA), and sh-GUSB+ anti-PD1 with 5 mice in each group. To be specific, the sh-NC+ anti-PD1 and sh-GUSB+ anti-PD1 groups received 200 ug of anti-PD1 intraperitoneal injection on the 8th day and then every three days.

Mice model Part ii: C57BL/6 mice were made by injecting Hepa1-6 cells. Cancer transplanted tumor model mice were divided into the control group, amoxapine group, anti-PD1 group, and amoxapine + anti-PD1 group, with 5 mice in each group. To be specific, the anti-PD1 or amoxapine+anti-PD1 group received a 200 ug anti-PD1 intraperitoneal injection on the 8th day and then every three days. Amoxapine or amoxapine+ anti-PD1 group received 5 mg/kg amoxapine intra-peritoneal injection on the 2nd day, and per two days thereafter. The control group received an intraperitoneal injection of PBS 100 ul/mouse on the second day and every two days thereafter. The activity, spirit, and diet of mice were observed per day before and after the experiment. The long diameter A (mm) and short diameter B (mm) of the tumor were decided by vernier calipers every four days, and the volume of tumor (V) of mice was counted by V = AB^2^/2, and the tumor growth curve could be plotted. After 20 days, mice could be sacrificed by neck dissection.

### Mass Spectrometry

Tissue samples were obtained from sh-NC and sh-GUSB mice, separately. The mouse tissue was treated with a Miltenyi Mouse Tumor Kit and Percoll to remove impurities and divide the red color. CyTOF staining steps consisted of 194Pt staining → Fc block → surface antibody staining → overnight DNA staining (191/193Ir) → intracellular antibody staining →computer data collection. The data analysis processes included a. FlowJo pretreatment: single, live, intact CD45^+^ immune cells were cyclically selected; and b. Biological information analysis: X-Shift algorithm for cell subspecies clustering, manual labeling, TSNE dimensional reduction visualization, and statistical analysis.

### Software Data Analysis

UALCAN (http://ualcan.path.uab.edu/cgi-bin/ualcan-res.pl) was used here to compare GUSB expression in HCC patients of different parts, tumor grades, and lymph node metastasis. The co-relationship among GUSB expression and immune elements was forecasted by the TISIDB database (http://cis.hku.hk/TISIDB/index.php).

### Statistical Analysis

GraphPad Prism 8.0 (GraphPad, USA) was used for statistical analysis, and a P-value <0.05 was considered statistically significant. We performed an independent t-test for continuous variable comparisons among two groups and a one-way ANOVA for comparisons between multiple groups.

## Results

### GUSB Was Significantly Upregulated in Anti-PD1 Non-Responding Tumors Compared With Responding Tumors in HCC Patients

**Figure 1:**
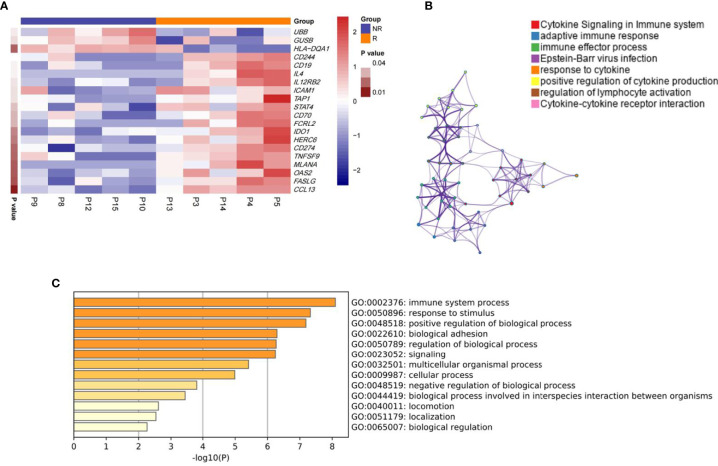
GUSB was significantly upregulated in anti-PD1 non-responding tumors compared with responding tumors in HCC patients **(A)** 10 HCC patients were treated with anti-PD1, and the biopsy samples before treatment were used for 289 nanostring panel RNA sequencing to compare responding (≥50% tumor necrosis) (R) and non-responding tumors (<50% tumor necrosis) (NR). Panel **(A)** is presented in the form of a heatmap. **(B, C)** Pathway analysis showing the different genes’ enrichment. Panel **(B)** is presented as a mesh graph and Panel **(C)** as a bar graph.

Ten HCC patients were treated with anti-PD1. Biopsy samples were sequenced with 289 nanostring panel RNA before treatment, and the response (≥50% tumor necrosis) (R) and non-response (<50% tumor necrosis) (NR) were compared (14). In contrast to non-responsive tumors, biopsy of responsive tumors showed higher transcription levels of CD244, CD274, CCL13, IDO1, CD70, IL12RB2, CD19, IL4, and lower transcription levels of UBB and GUSB (**Figure 1A**). A pathway study indicated that the different genes were primarily enriched in cytokine signaling in the immune system, adaptive immune response, etc. (**Figures 1B, C**). We also analyzed the PD1, PD-L1, HAVCR2, TIGIT, CTLA4, and GUSB expression in HCC biopsy samples and found that GUSB expression was negatively associated with the gene expression mentioned above (**Figures S1A–E**). TISIDB database also showed that GUSB expression was negatively associated with PD1, HAVCR2, TIGIT, and CTLA4 expression in 373 HCC samples (**Figures S2A, B**). Additionally, the UALCAN database revealed that GUSB expression in tumor tissues was clearly higher than that in normal tissues (**Figure S3A**). The subgroup study according to stages, tumor grade, and lymph node metastasis showed that the higher cancer stage, grades, and lymph node metastasis, the higher GUSB expression (**Figures S3B–D**). Therefore, the results showed that GUSB could be a vital factor in reducing PD-L1 expression and inhibiting the immune response in HCC anti-PD1 therapy.

### Knocked Down of GUSB Promoted the CD8^+^ T Cell Exhaustion Markers Expression When Co-Cultured With Antigen Specific CD8^+^ T Cells Separated From PBMC Specimens

**Figure 2:**
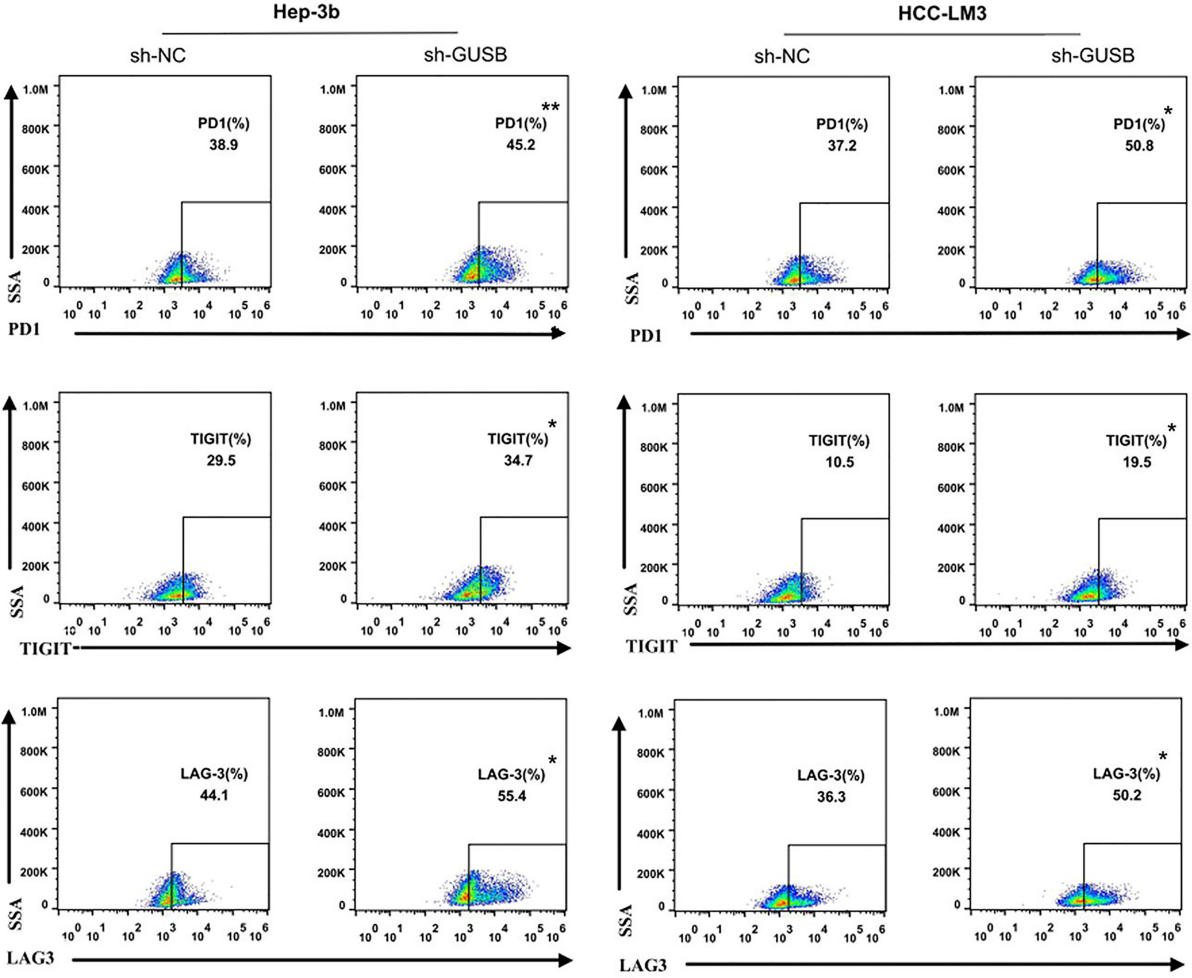
Knocked down of GUSB promoted the CD8^+^ T-cell exhaustion markers expression when co-cultured with antigen-specific CD8^+^ T cells isolated from 3 PBMC samples. HCC cells treated with sh-NC/sh-GUSB were co-cultured with antigen-specific CD8^+^ T cells, and the expression of common markers of CD8^+^ T-cell exhaustion (LAG3, PD1, and TIGIT) was measured by flow cytometry. *P < 0.05; **P < 0.01.

To investigate the effect of GUSB on the immune microenvironment of HCC *in vitro*, human HCC cells (Hep-3B, HCC-LM3) were co-cultured with sh-GUSB or sh-NC, and antigen-specific CD8^+^ T cells isolated from 3 PBMC specimens were co-cultured. Flow cytometry was used to detect the expression of immune factors. The results showed that common exhaustion markers of CD8^+^ T cells (LAG3, PD1, and TIGIT) were evidently increased (**Figures 2**, **S4**). These results showed that GUSB knockout promoted the expression of CD8^+^ T cell exhaustion markers when co-cultured with antigen-specific CD8^+^ T cells separated from PBMC of HCC patients.

### Knocked Down of GUSB Inhibited the Proliferation, Invasion and Migration of HCC Cells and Upregulated PD-L1 Expression *Via* Decreasing miR-513a-5p

**Figure 3:**
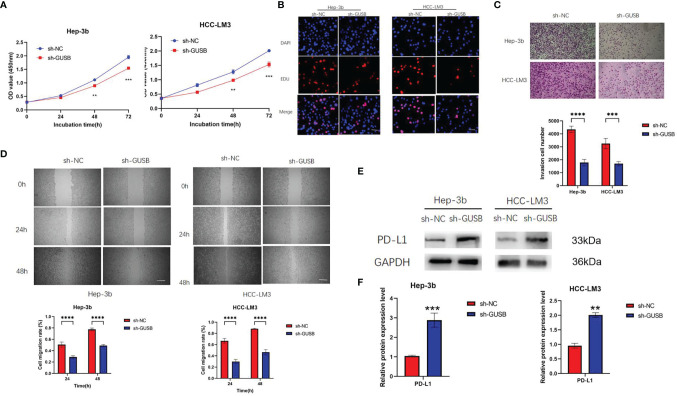
Knocked down of GUSB inhibited the proliferation, invasion and migration of human HCC cells and up-regulated PD-L1 expression. **(A)** The growth curves of cells were plotted after transfection with sh-NC/sh-GUSB based on CCK-8 assay. **(B)** EdU assay was performed to assess cell proliferation of HCC cells transfected with sh-NC/sh-GUSB. Scar bar, 50 um. **(C)** Transwell invasion assay was performed to assess invasion of HCC cells transfected with sh-NC/sh-GUSB. Scar bar, 100 um. **(D)** Scratch assay was performed to assess the migration of HCC cells transfected with sh-NC/sh-GUSB. Scar bar, 250 um. **(E, F)** The protein expression of PD-L1 in HCC cells with GUSB knockdown. Panel **(E)** represents the variation of protein bands, and Panel **(F)** illustrates the protein gray value analysis. Each experiment was repeated three times. **P < 0.01; ***P < 0.001; ****P < 0.0001.

shRNA against GUSB (sh-GUSB) was developed to silence GUSB in Hep-3b and HCC-LM3 cells. Results showed that sh-GUSB inhibited proliferation in Hep-3b and HCC-LM3 cells, as per the consequences of CCK-8 and EdU tests (**Figures 3A, B**). A transwell invasion assay indicated that sh-GUSB remarkably prevented the invasion functions of Hep-3b and HCC-LM3 cells (**Figure 3C**). Scratch tests showed that the migration ability significantly weakened HCC cells knocked down by GUSB (**Figure 3D**). Additionally, a western blot assay was used to verify that downregulated GUSB led to increased PD-L1 expression in HCC cells (**Figures 3E, F**), which was consistent with 289 Nanostring panel RNA sequencing.

**Figure 4:**
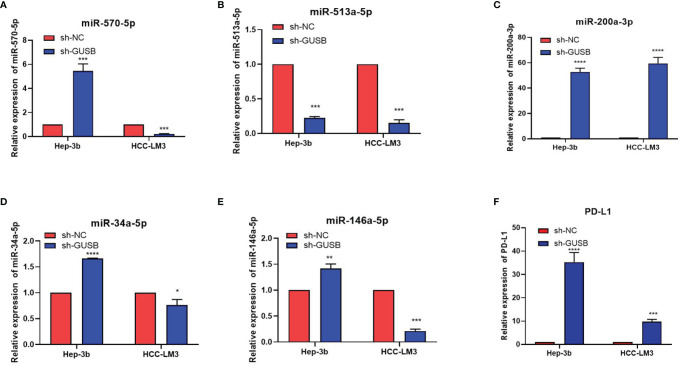
Knocked down of GUSB upregulated PD-L1 expression by decreasing miR-513a-5p expression. **(A–E)** qRT-PCR results of the changes of miRNAs caused by sh-GUSB to HCC cells. **(F)** qRT-PCR results for PD-L1 in HCC cells. *P <  0.05; **P < 0.01; ***P < 0.001; ****P < 0.0001.

We attempted to investigate the influencing factors of the PD-L1 increase in HCC cells caused by sh-GUSB. MicroRNAs (miRNAs) are 21–23 nt single-stranded regulatory RNA molecules, which monitor the expression of genes on the basis that they complement the 3′-untranslated region of target mRNA and result in mRNA cleavage and/or translation inhibition. After reading many documents, we selected miR-570-5p, miR-513a-5p, miR-200a-3p, miR-34a-5p, and miR-146a-5p as testing objects (15–21). qRT-PCR was used to examine these miRNAs expression in HCC cells cultured with sh-NC or sh-GUSB and the results indicated that miR-513a-5p expression was both downregulated in Hep-3b and HCC-LM3 cells, whereas miR-570-5p, miR-200a-3p, miR-34a-5p, and miR-146a-5p were not expressed as expected (**Figures 4A–E**). Additionally, mRNA of PD-L1 was upregulated in HCC cells cultured *via* sh-NC or sh-GUSB (**Figure 4F**).

The knockdown of GUSB was also verified in mouse HCC cells-Hepa1-6. We were pleasantly surprised to find that miR-513a-5p decreased and PD-L1 increased after GUSB knockdown, which was consistent with the results in human HCC cells (**Figure S5**). Gong et al. confirmed that miR-513a-5p regulates PD-L1 and is involved in IFN-γ-induced PD-L1 expression in human cholangiocytes. Transfection of cholangiocytes with an antisense oligonucleotide to miR-513a-5p induced PD-L1 protein expression. miR-513a-5p targeted a potential binding site in the PD-L1 3′-UTR resulting in translational suppression (15). These results demonstrated that the downregulation of GUSB prevented the proliferation, invasion, and migration of human HCC cells and upregulated PD-L1 expression by decreasing miR-513a-5p.

### Knock Down of GUSB Enhanced the Anti-HCC Effect When Combined With Anti-PD1 Therapy *In Vivo*

**Figure 5:**
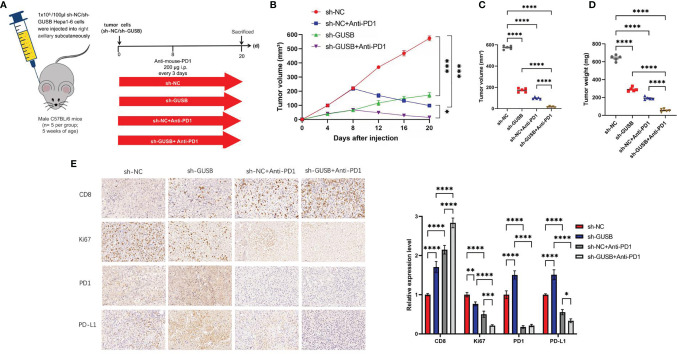
Knockdown of GUSB enhanced the anti-HCC effect when combined with anti-PD1 therapy *in vivo*. **(A)** Schematic diagram of animal experiment. We injected Hepa1-6 cells (sh-NC, sh-GUSB, sh-NC + anti-PD1, and sh-GUSB + anti-PD1) into C57BL/6 mice. **(B–D)** The volume **(B, C)** and weight **(D)** statistics of subcutaneous tumors in the respective groups (sh-NC, sh-GUSB, sh-NC + anti-PD1, sh-GUSB + anti-PD1). **(E)** Immunohistochemistry result of CD8, Ki67, PD1, and PD-L1 expression in the respective groups. Scar bar, 50 um. *P < 0.05; **P < 0.01; ***P < 0.001; ****P < 0.0001.

To address the possible influence of GUSB on the tumor immune response, Hepa1-6 cells (sh-NC, sh-GUSB, sh-NC+anti-PD1, and sh-GUSB+anti-PD1) were injected into C57BL/6 mice (**Figure 5A**). Hepa1-6 cells with sh-NC grew rapidly in mice, while they attenuated in sh-GUSB mice (**Figures S6**, **5B**). When anti-PD1 was added on day 8, tumor growth tended to be slow (**Figure 5B**). On day 20, the tumor volume and weight of the sh-GUSB group were significantly smaller than those of the sh-NC group, and the tumor volume and weight of the sh-GUSB +anti-PD1 group were significantly smaller than those of the sh-GUSB group and sh-NC +anti-PD1 group (**Figures 5C, D**). Based on immunohistochemistry consequences, compared to the sh-NC group, PD1, PD-L1, and CD8 expression were significantly increased after GUSB knockdown, while the expression of Ki67 was decreased. In the sh-GUSB+ anti-PD1 group, CD8 was much more highly expressed, and Ki67, PD-L1, and PD1 were significantly decreased (**Figure 5E**).

**Figure 6:**
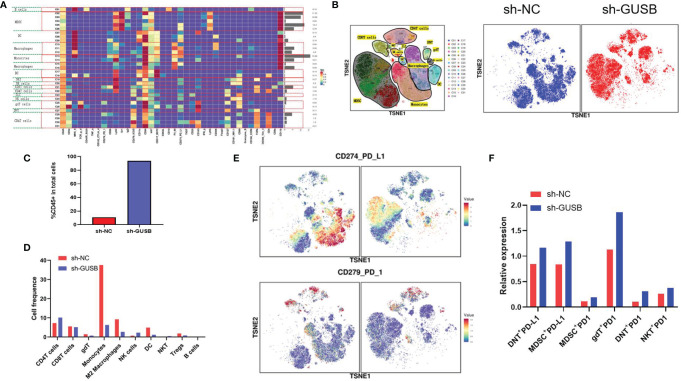
Mass cytometry reflected the immune microenvironment of HCC after sh-NC/sh-GUSB treatment. **(A)** There were 31 cell clusters in total, which were defined in the respective groups. **(B)** TSNE plot showing the distribution of 31 cell clusters and showing the distribution of cell clusters in the respective samples. **(C)** The histogram shows the number of CD45 ^+^ immune cells in each group. **(D)** The histogram shows the number of the respective cell clusters in different groups by mass cytometry. **(E)** TSNE plot showing the distribution of PD1/PD-L1 in two groups. **(F)** The histogram shows the number of PD1^+^ cell clusters in different groups.

Based on mass spectrometry, we explored the immunization infiltration of Hepa1-6 tumors (sh-NC, sh-GUSB) within mice 20 days after tumors were injected. We selected cells from their respective tissues and recycled single, live, and intact CD45^+^ immune cells. All specimens indicated clustering and subgroup annotation in CD45^+^ immune cells. There were 31 cell clusters, and the corresponding cell clusters were defined according to specific markers of cell types (**Figures 6A, B**, **S7**). As indicated by the result, the expression level of CD45*^+^* immune cells was significantly increased in the sh-GUSB part compared to the sh-NC part (**Figure 6C**).

Relative proportions of M2 macrophages, monocytes, DC cells, and Tregs in the sh-GUSB group showed a downward trend compared to the sh-NC part, whereas CD4*^+^* T and NK cells showed an increasing trend (**Figure 6D**). CD8*^+^* T cells did not indicate an obvious change (**Figure 6E**). Additionally, it was shown that DNT ^+^ PD-L1^+^, MDSC^+^ PD-L1^+^, gDT^+^ PD-L1^+^, DNT^+^ PD-L1^+^, and NKT^+^ PD-L1^+^ cell expression increased in the sh-GUSB part compared with the sh-NC part (**Figures 6E, F**). These consequences confirmed that knockdown of GUSB enhanced the anti-HCC effect when combined with anti-PD1 therapy *in vivo*.

### Amoxapine Decreased the Proliferation, Invasion and Migration of Human HCC Cells *Via* Inhibiting GUSB and Upregulated PD-L1 Expression

**Figure 7:**
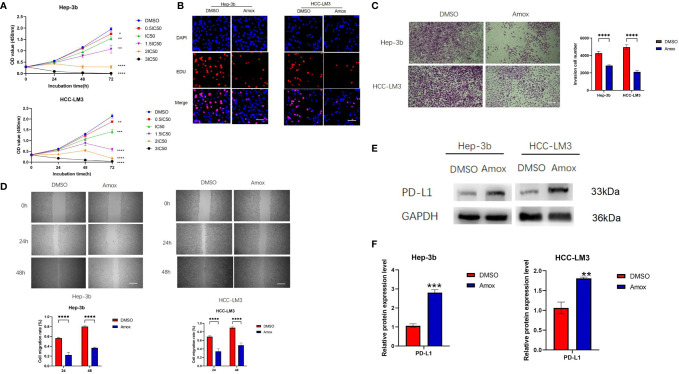
Amoxapine decreased the proliferation, invasion, and migration of human HCC cells by inhibiting GUSB and upregulating PD-L1 expression in HCC. **(A)** The growth curves of cells were plotted after adding DMSO or amoxapine based on the CCK-8 assay. **(B)** EdU assay was performed to assess cell proliferation of HCC cells added with DMSO or amoxapine. Scar bar, 50 um. **(C)** Transwell invasion assay was performed to assess invasion of HCC cells added with DMSO or amoxapine. Scar bar, 100 um. **(D)** A Scratch assay was performed to assess the migration of HCC cells added with DMSO or amoxapine. Scar bar, 250 um. **(E, F)** The protein expression of PD-L1 in HCC cells was added with DMSO or amoxapine. Panel **(E)** represents the variation of protein bands, and Panel **(F)** illustrates the protein gray value analysis. Each experiment was repeated three times. *P < 0.05; **P < 0.01; ***P < 0.001; ****P < 0.0001.

Previous literature has reported that the older drug amoxapine could act as an effective GUSB inhibitor to reduce tumor growth (22). Therefore, we discussed the anticancer effect of amoxapine on HCC and the sensitization to anti-PD1 therapy. Surprisingly, we found that the addition of amoxapine significantly reduced the proliferation, invasion, and migration of HCC cells (**Figures 7A–D**). Additionally, the western blot method was used to verify that the addition of amoxapine in HCC cells could increase the expression of PD-L1 (**Figures 7E, F**), which was consistent with the result of GUSB knockdown.

### Amoxapine Worked as an Effective Means to Treat HCC and Improved the Sensitivity of Anti-PD1 Therapy

**Figure 8:**
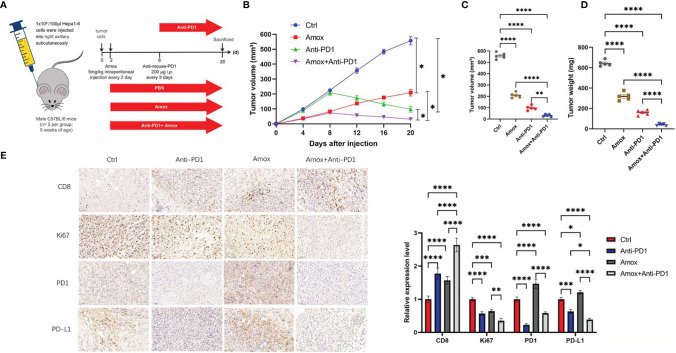
Amoxapine worked as an effective means to treat HCC and improved the sensitivity of anti-PD1 therapy. **(A)** Schematic diagram of animal experiment. we injected Hepa1–6 cells in C57BL/6 mice. **(B–D)** The volume **(B, C)** and weight **(D)** statistics of subcutaneous tumors in the respective groups (PBS, amoxapine, anti-PD1, amoxapine + anti-PD1). **(E)** Immunohistochemistry result of CD8, Ki67, PD1, and PD-L1 expression in the respective groups. Scar bar, 50 um. *P < 0.05; **P < 0.01; ***P < 0.0001; ****P < 0.0001.

Whether amoxapine can inhibit tumors and enhance anti-PD1 sensitivity *in vivo* is worth further exploration. The Hepa1-6 cells (PBS, amoxapine, anti-PD1, amoxapine + anti-PD1) were injected into C57BL/6 mice to investigate the possible effect of amoxapine on tumor immune response (**Figure 8A**). Compared with the PBS group, Hepa1-6 cells in the amoxapine group tended to slow down, whereas attenuated cells in the amoxapine + anti-PD1 group tended to increase (**Figures S8**, **8B**). When the mice were sacrificed on day 20, the volume and weight of the tumor in the amoxapine portion were found to be remarkably smaller than those in the PBS group, and those in amoxapine + anti-PD1 were much smaller than those in the amoxapine or anti-PD1 groups (**Figures 8C, D**). Based on immunohistochemistry consequences, compared to the sh-NC group, PD1, PD-L1, and CD8 expression were increased after amoxapine was added, whereas the expression of Ki67 was decreased. In the amoxapine+anti-PD1 group, CD8 expression was much higher, as well as Ki67, PD-L1, and PD1 expression were remarkably decreased (**Figure 8E**). All of these results indicate that amoxapine could work as an effective means to treat HCC and improve the sensitivity of anti-PD1 therapy.

## Discussion

In the tumor microenvironment, tumor cells and antigen-presenting cells (APCs) upregulate PD-L1 expression to evade immune surveillance through adaptive and internal immune tolerance. Furthermore, the increase in PD-L1 is also a prerequisite for the effectiveness of anti-PD1/PD-L1 monoclonal antibodies (7). In murine experiments, Juneja et al. showed that the expression level of PD-L1 on tumors and that immune cells play a vital role in the effect on PD1 antibodies (23). Thus, PD1/PD-L1 expression can be impacted by regulating many of the aforementioned regulators to improve the response to PD1/PD-L1 inhibitors in patients with or without tumors. In our study, we demonstrated that PD-L1 was significantly declined in unresponsive tumors compared with those that were positive in anti-PD1 responses in HCC patients, which is consistent with a previous study (7). Additionally, to analyze which genes caused primary drug resistance to anti-PD1 treatment, we sequenced 289 nanostring panel RNA and found increased GUSB expression in the anti-PD1 resistance group, thus finding new predictive molecules and therapeutic targets for anti-PD1 therapy in HCC.

GUSB is a key lysosomal enzyme related to the degradation of glucuronate glycosaminoglycan. The lack of GUSB leads to mucopolysaccharide storage disease type VII, which causes lysosomal storage in the brain. The expression, sequence, structure, and function of GUSB are worthy of study (8). This study elucidates the key role of intestinal bacterium GUSB in the pathogenesis of diarrhea caused by irinotecan. Intestinal GUSB hydrolyzes glucuronide metabolites into the toxic style of the intestine, causing intestinal damage. Selected inhibitors of GUSB presently under development may relieve diarrhea caused by irinotecan and can contribute to decreasing the incidence and altering the activity (24, 25). However, there are not many studies on GUSB directly against cancer. Bhatt et al. demonstrated that targeted inhibition of gut microbial GUSB enzymes protected the digestive tract from epithelial cell toxicity in pre-clinical models. GUSB inhibition can significantly improve human cancer therapy with irinotecan and various glucuronidating chemotherapeutic drugs that cause intestinal toxicity and reduce tumor size (12). Kong et al. have reported that amoxapine and its metabolites are bound to the active site of GUSB, satisfying the key pharmacophore characteristics. Low dose amoxapine plus irinotecan significantly inhibits diarrhea and tumor growth in mice (22). In our study, we found that knocking down of GUSB promoted the CD8^+^ T-cell exhaustion marker expression co-cultured with antigenspecific CD8^+^ T cells separately from PBMC specimens, and down-expression of GUSB prevented proliferation, invasion, and migration of human HCC cells and upregulated PD-L1 expression by decreasing miR-513a-5p expression. The knockdown of GUSB enhanced the anti-HCC effect when combined with anti-PD1 therapy *in vivo*, which adds luster to the new function of GUSB in cancer.

Notably, we studied the immune invasion of Hepa1-6 tumors (sh-NC, sh-GUSB) in mice 20 days after tumor injection by mass spectrometry. As indicated from the result, the expression level of CD45*^+^* immune cells was remarkably increased in the sh-GUSB part compared to the sh-NC part, suggesting that the reduction of GUSB would massively activate tumor immune infiltration. The relative proportion of M2 macrophages, monocytes, DC cells, and Tregs in the sh-GUSB group showed a downward trend compared with the sh-NC group, whereas CD4*^+^* T and NK cells indicated an increasing trend. There was no evident change in CD8^+^ T cells, which may have been caused by the insignificant sample size or insufficient mass spectrometry grouping, but our immune-histochemical results further confirmed that the reduction of GUSB significantly increased the infiltration of CD8^+^ T cells.

Additionally, it was discovered that DNT ^+^ PD-L1^+^, MDSC^+^ PD-L1^+^, gDT^+^ PD-L1^+^, DNT^+^ PD-L1^+^, and NKT^+^ PD-L1^+^ cell expression increased in the sh-GUSB group compared with the sh-NC part. Based on this, a combination of PD1 mAb will be more effective in killing HCC cells. Therefore, the central idea of this study is that the high expression of GUSB leads to insensitivity to anti-PD1 therapy in HCC patients. High expression of GUSB in HCC cells can lead to: 1) promoting cancer cell growth; 2) reducing PD-L1 expression, which is less responsive to anti-PD1 therapy; and 3) promoting immunosuppression, making HCC patients more inclined to a desert-type tumor immune microenvironment, which has a negative impact on anti-PD1 response. Therefore, knockdown of GUSB in cancer cells can 1) inhibit the growth of cancer cells, 2) increase NK and CD8^+^ T cells in the tumor microenvironment and decrease immunosuppressive cells such as Tregs and M2 macrophages. However, the side impact on the knockdown of GUSB is to increase the exhaustion factors such as PD-L1, PD1, TIGIT, etc., but the increase in exhaustion factors was not sufficient to resist the killing effect of knocking down GUSB. In the tumor immune microenvironment with abundant CD8^+^ T and NK cells, low expression of Treg and M2 macrophages, and high expression of PD1, combined anti-PD1 may be more beneficial in HCC patients.

The biggest highlight of this research is that we reported that amoxapine acting as a GUSB inhibitor decreased proliferation, invasion, and migration of human HCC cells by upregulating PD-L1 expression. Amoxapine could work as an effective means to treat HCC and improve the sensitivity of anti-PD1 therapy. We have not only found a new target of anti-PD1 resistance but also found an effective way to inhibit this target by using new methods of old drugs like amoxapine. Even as an old drug whose properties and toxicity are widely recognized, amoxapine still has good underpinning for clinical application to be moved to human subjects because of its ability to prevent irinotecan-induced diarrhea with minimal side effects and improve the effectiveness of chemotherapy. Our study provides a new approach for enhancing anti-PD1 therapy in HCC patients.

There were some limitations to our study. Firstly, although we demonstrated that the reduction of GUSB may increase PD-L1 by reducing the expression of miR-513a-5p, its specific molecular site was not further elucidated. Secondly, due to financial constraints, we did not detect three samples in the mass spectrometry section, but we further verified our conclusions by immunohistochemistry. Thirdly, before anti-PD1 treatment, we did not further verify the expression of GUSB in human samples because the size of the puncture sample was too small for further verification.

## Conclusion

**Figure 9:**
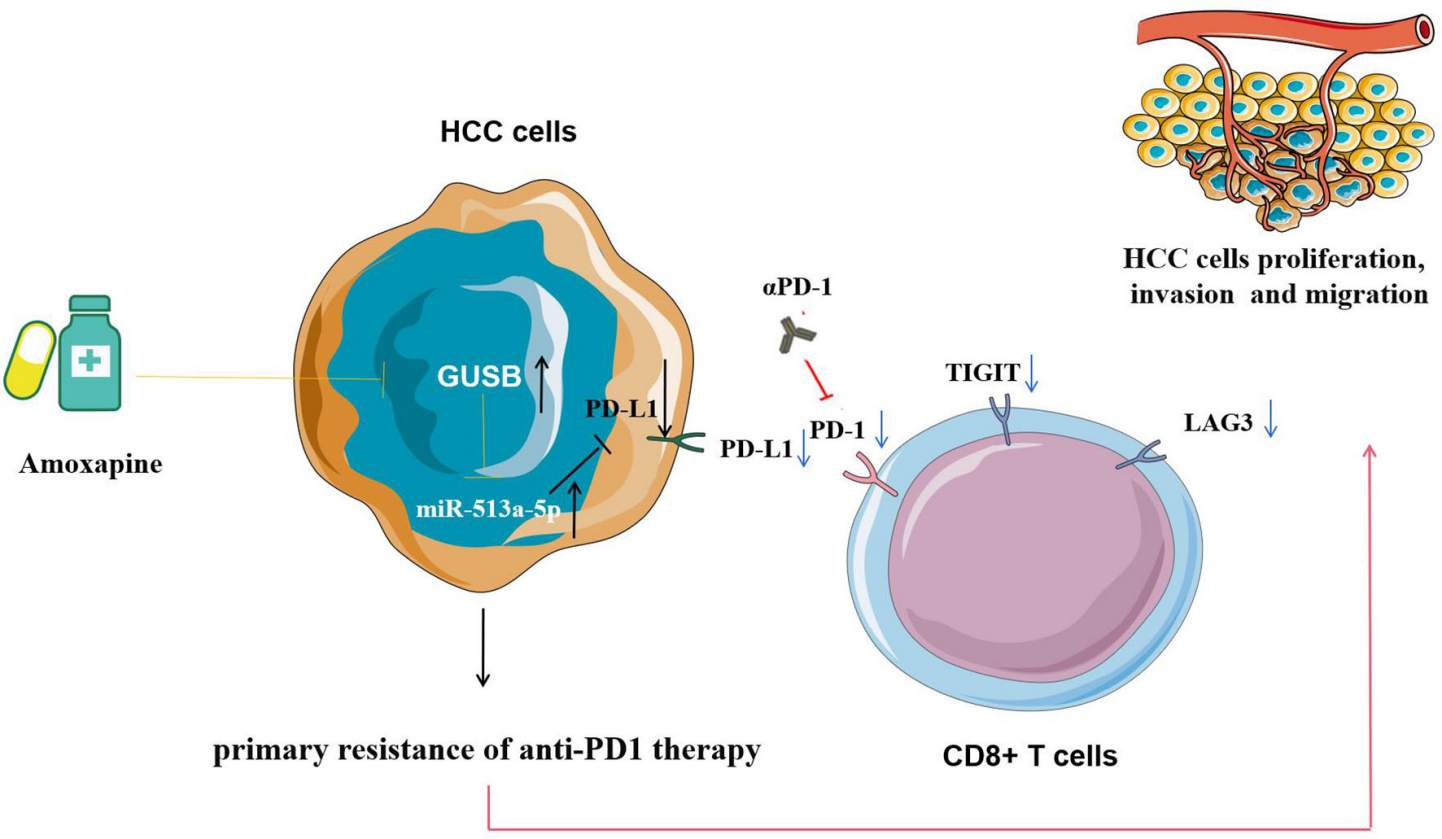
Pattern diagram showing that increased GUSB inhibits the expression of PD-L1 by promoting miR-513a-5p expression, leading to primary resistance to anti-PD1 therapy in HCC, and the use of amoxapine enhances the sensitivity of anti-PD1 therapy by inhibiting GUSB in HCC.

In summary, this study reveals that increased GUSB inhibits the expression of PD-L1 by promoting miR-513a-5p, leading to primary resistance to anti-PD1 treatment in HCC, and the use of amoxapine enhances the sensitivity of anti-PD1 treatment by inhibiting GUSB in HCC (**Figure 9**). This research offers a novel strategy and method for improving the therapeutic effect of anti-PD1 therapy and brings new hope for treating HCC.

## Data Availability Statement

The data presented in the study are deposited in the NCBI repository, accession number GSE198190.

## Ethics Statement

The animal study was reviewed and approved by the Animal Management Committee of Nanjing Medical University.

## Author Contributions

XK, ZYZ, and GXS were responsible for designing and performing the experiments. ZHZ and HL were responsible for the manuscript language editing. JK, GGS, GSS, TH, XL, and DR contributed to performing part of the experiment. KW, WT, and YX have contributed to data interpretation, editing and critical revision of the manuscript. WT and YX have contributed to the study design and critical revision of the manuscript. All authors listed have made a substantial, direct, and intellectual contribution to the work and approved it for publication.

## Funding

We are grateful for the grants from the National Natural Science Foundation of China (No. 82070676) and the National Natural Science Youth Foundation of China (No. 81771716).

## Conflict of Interest

The authors declare that the research was conducted in the absence of any commercial or financial relationships that could be construed as a potential conflict of interest.

## Publisher’s Note

All claims expressed in this article are solely those of the authors and do not necessarily represent those of their affiliated organizations, or those of the publisher, the editors and the reviewers. Any product that may be evaluated in this article, or claim that may be made by its manufacturer, is not guaranteed or endorsed by the publisher.
