# Supplementary material for: Over-Expression of GUSB Leads to Primary Resistance of Anti-PD1 Therapy in Hepatocellular Carcinoma
Source: Front Immunol. 2022 Jun 22;13:876048. doi: 10.3389/fimmu.2022.876048 (PMC9257027; doi:10.3389/fimmu.2022.876048)

Supplementary figure legends:

Figure S1 (A-E) The expression of PD1, PD-L1, HAVCR2, TIGIT, CTLA4 and GUSB was analyzed in HCC tissues.

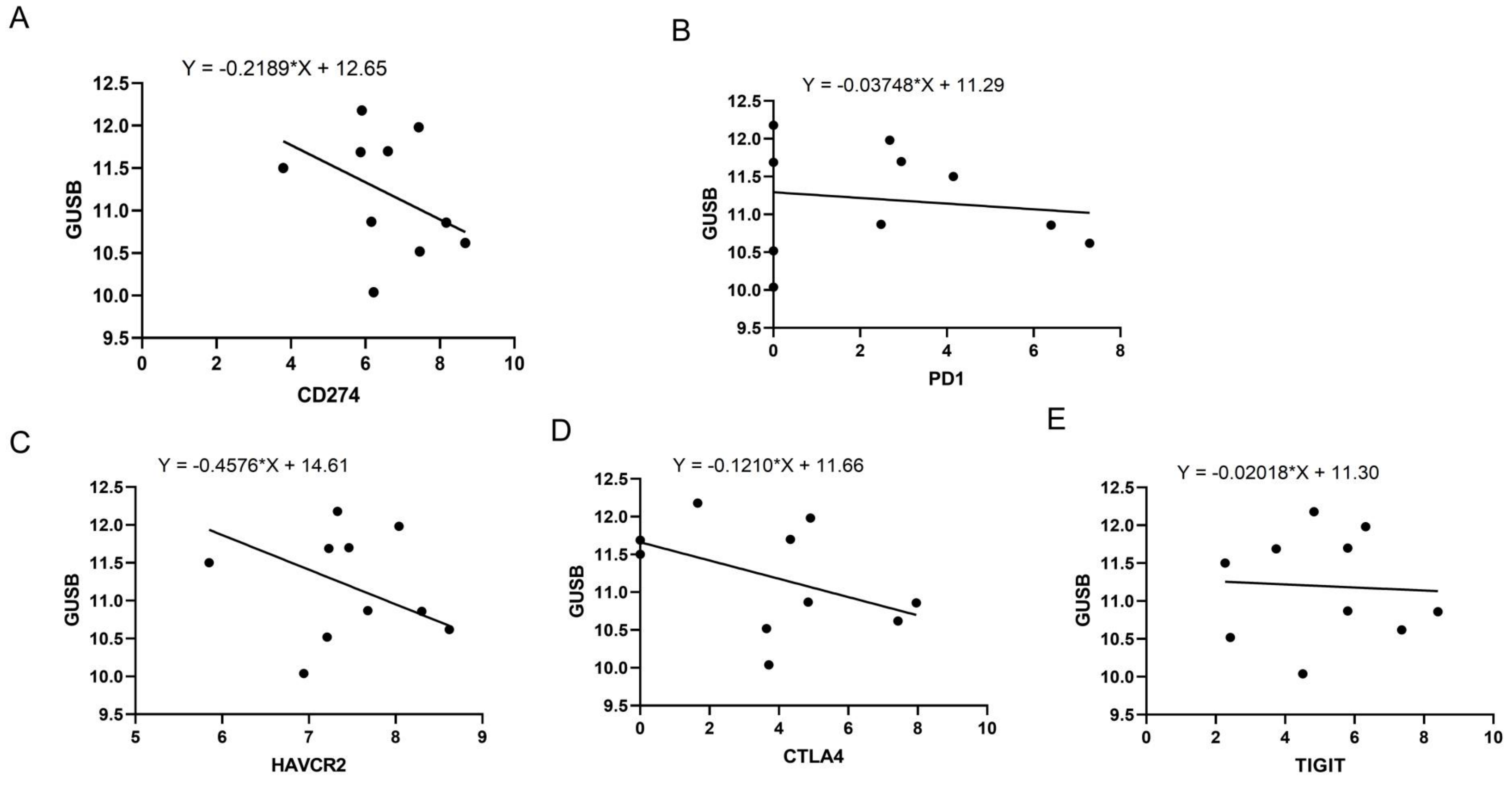

Figure S2 Correlation between GUSB expression and immunosuppressive cell expression. (A)The TISIDB database prediction indicated correlation between GUSB expression and the expression of immunoinhibitors.(B-D)The expression of GUSB was negative correlated with the expression of PD1, HAVCR2, TIGIT, and CTLA4 in 373 HCC samples.

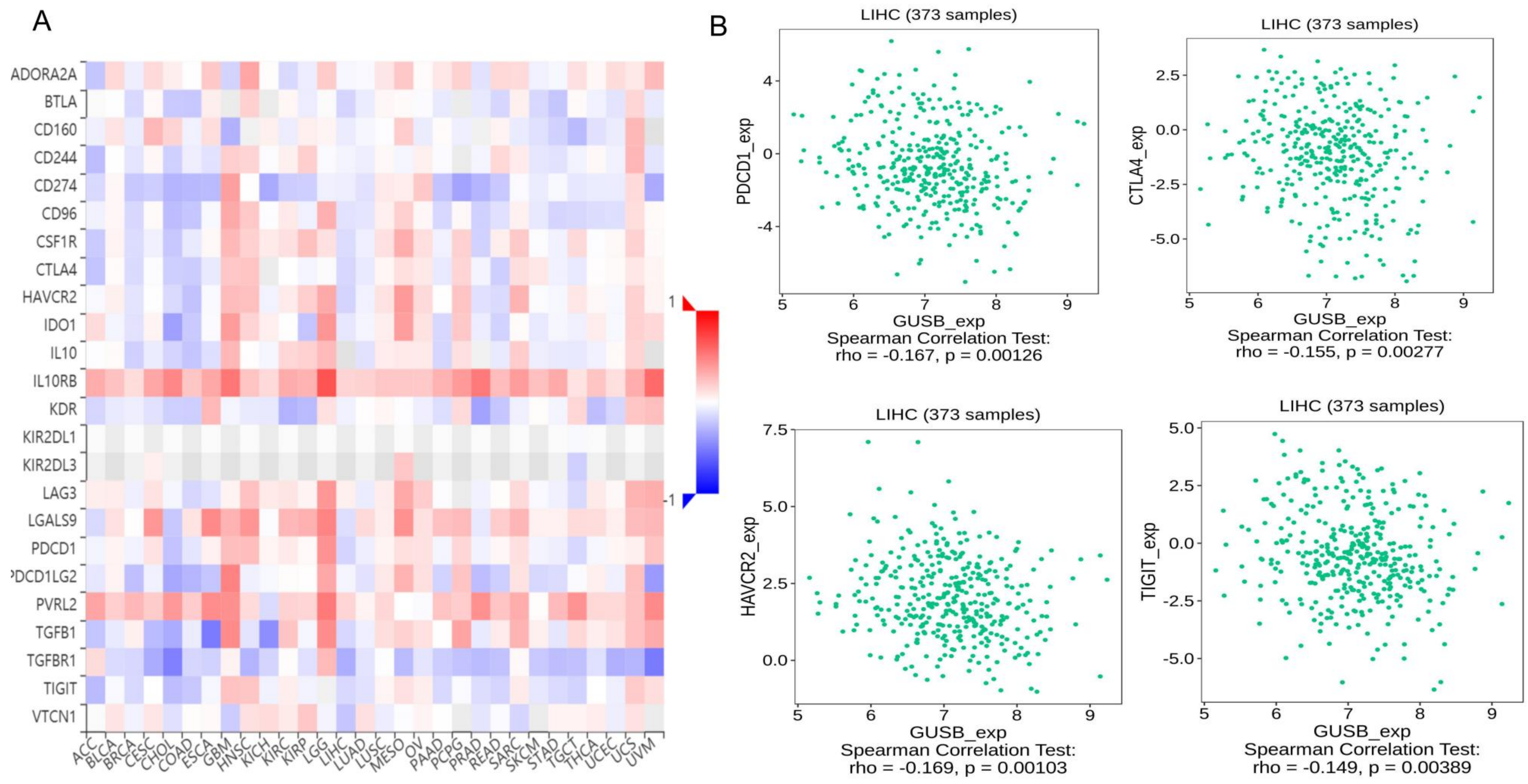

Figure S3 (A-F)The correlation between GUSB mRNA expression and HCC sample types, tumor stage,tumor grades, and lymph node metastatic status. *, $P < 0.05$; **, $P < 0.01$; ***, $P < 0.001$.

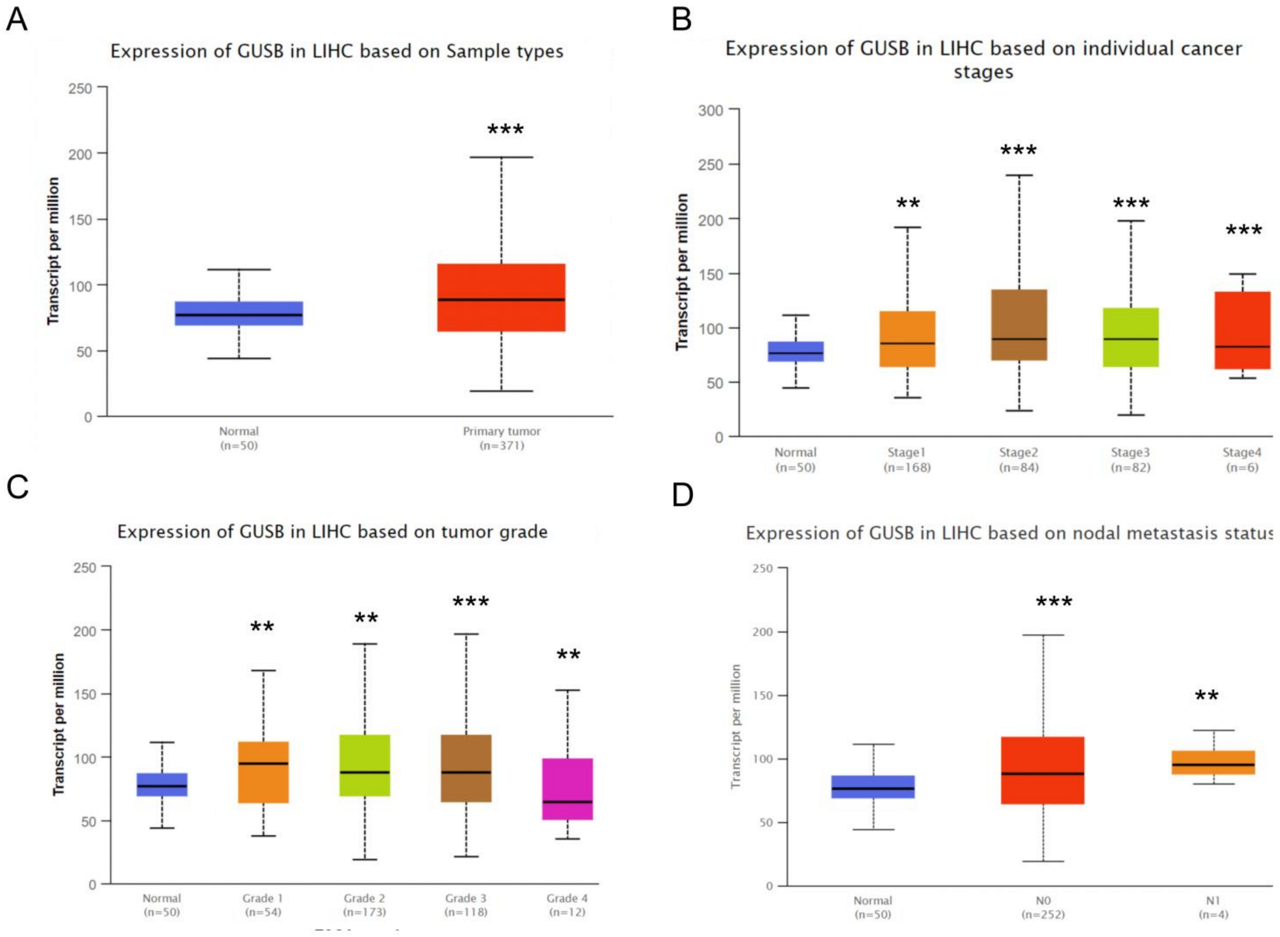

Figure S4 Comparison of blank tubes in flow cytometry.

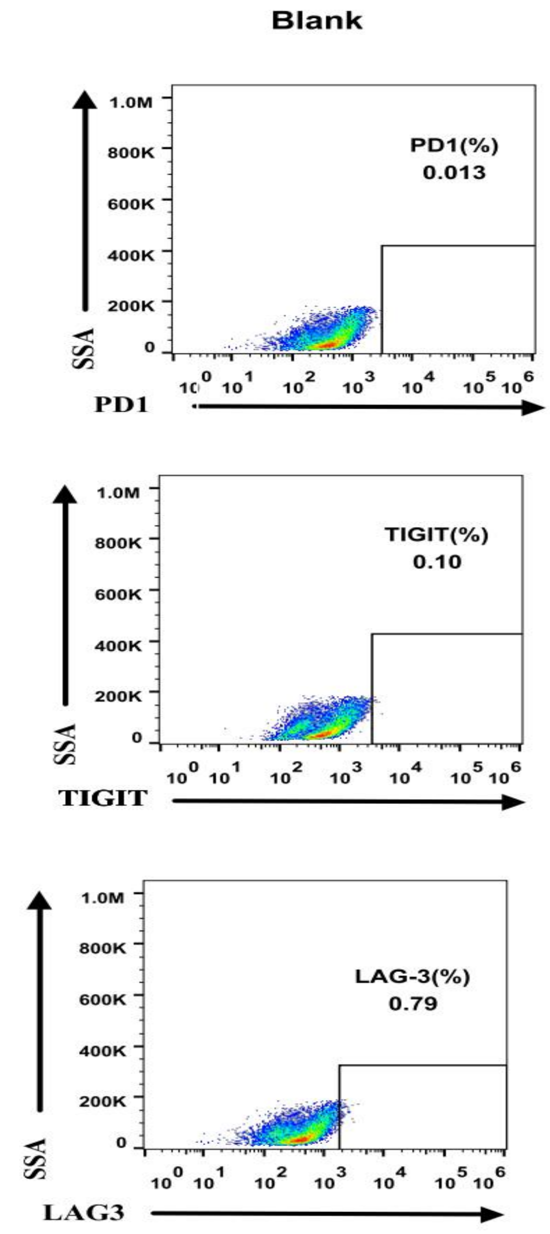

Figure S5 The expression of GUSB,miR-513a-5p and PD-L1 after GUSB knockdown in mice HCC cells-Hepa1-6.

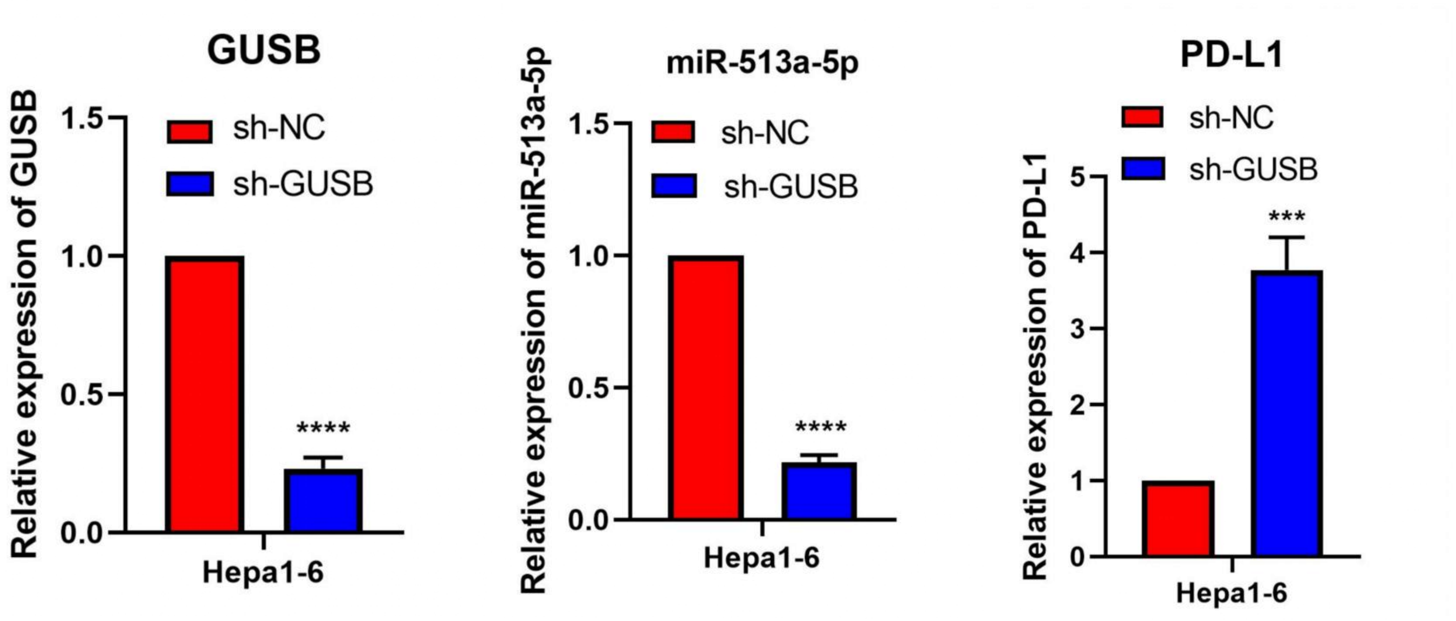

Figure S6 Illustration of mouse tumor in sh-NC, sh-GUSB, sh-NC+anti-PD1, sh-GUSB+anti-PD1 groups.

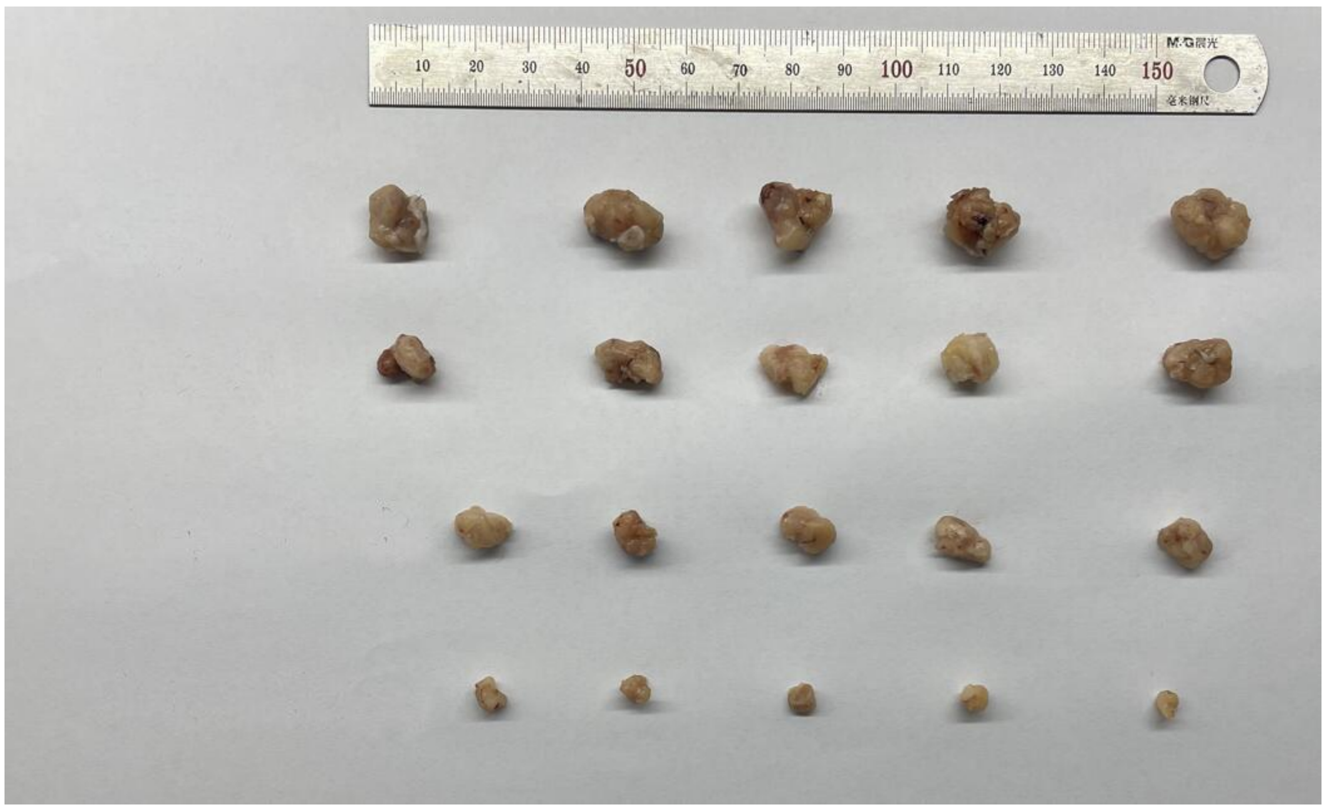

Figure S7 The expression of cell clustering maker genes measured by mass cytometry and presented in the form of TSNE plot.

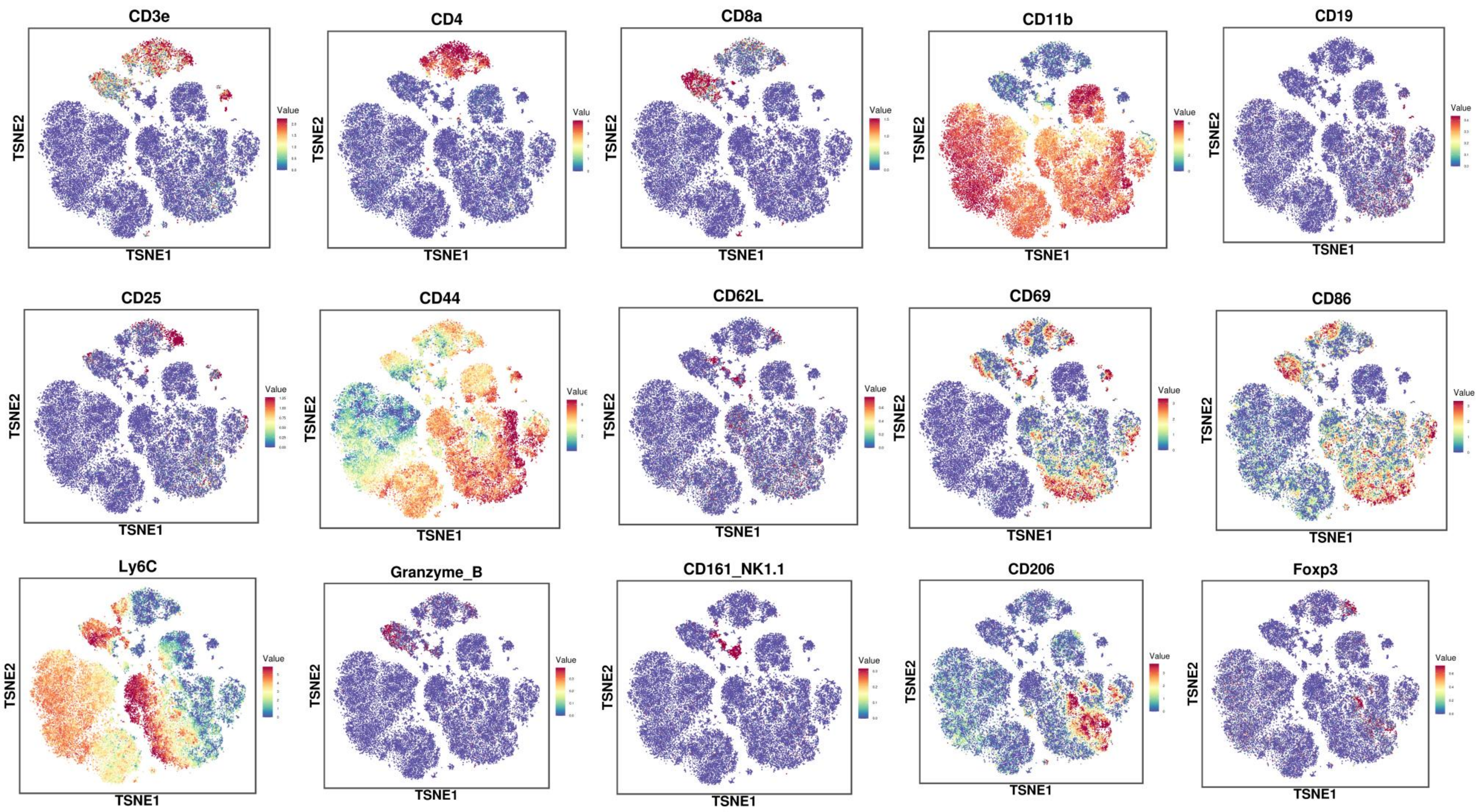

Figure S8 Illustration of mouse tumor in PBS, amoxapine, anti-PD1, amoxapine + anti-PD1 groups.

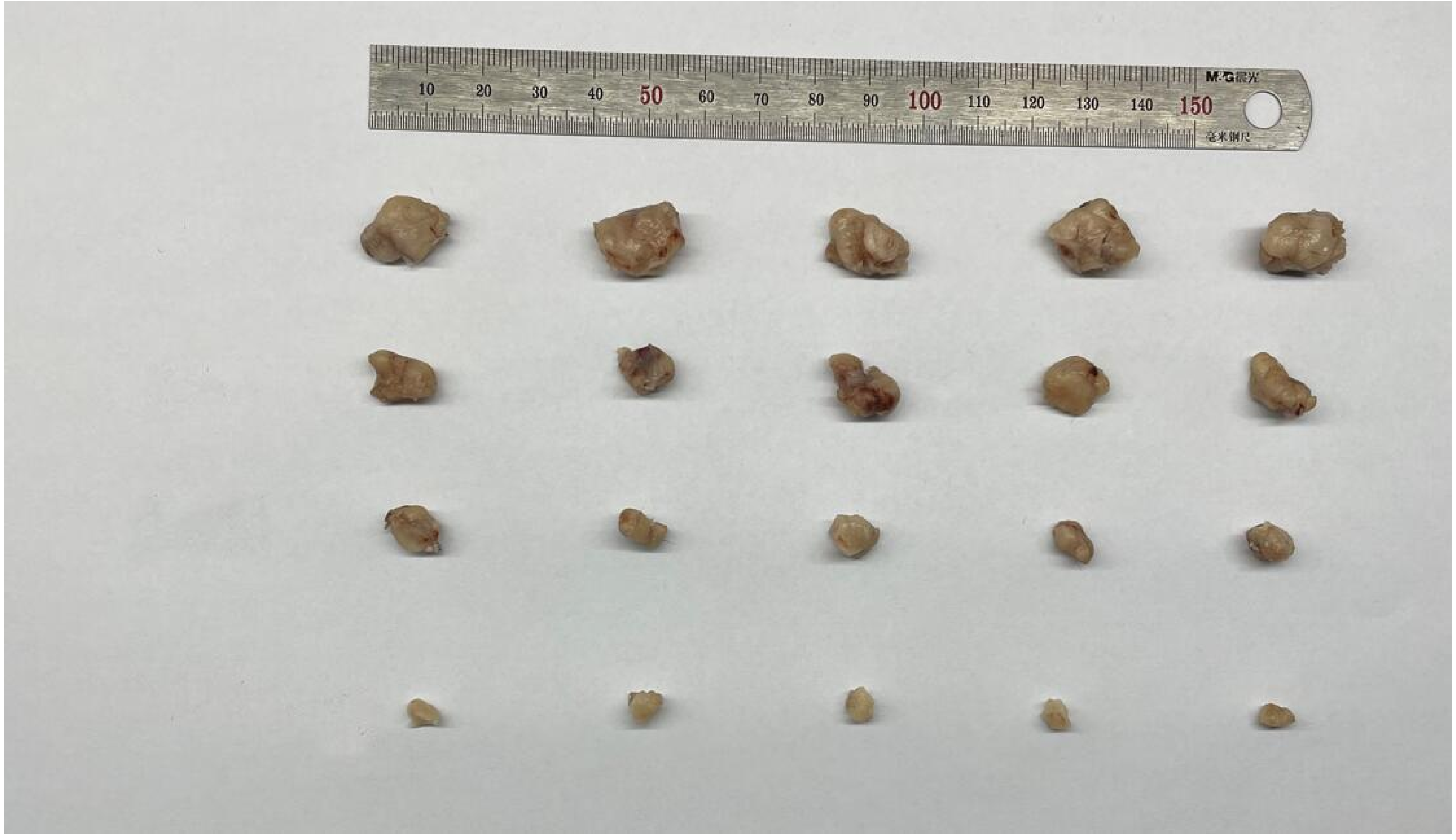

Supplement: Supplementary file 1 [file DataSheet_1.pdf]
